# Impact of Glucagon-Like Peptide 1 (GLP-1) and Gastric Inhibitory Polypeptide (GIP) Receptor Agonists on Intraocular Pressure in Glaucoma: A Case Series

**DOI:** 10.46889/joar.2026.7306

**Published:** 2026-09-27

**Authors:** Coy Steel, Catherine Johnson, Chase Paulson, Jesse Terrell, Qi N Cui, Lauren Blieden, Barbara Wirostko

**Affiliations:** 1 John A. Moran Eye Center, University of Utah, Salt Lake City, UT, USA; 2 Baylor College of Medicine, Houston, TX, USA; 3 Scheie Eye Institute, Perelman School of Medicine, University of Pennsylvania, Philadelphia, PA, USA

**Keywords:** Glaucoma, GLP-1 Receptor Agonists, GIP Receptor Agonists, Intraocular Pressure, Home Tonometry

## Abstract

**Purpose:**

Glucagon-Like Peptide 1 Receptor Agonists (GLP-1RAs) are antidiabetic and weight management medications that bind to GLP-1 receptors throughout the body. Growing interest in this class has led to several retrospective cohort studies reporting a decreased occurrence of glaucoma in diabetic and non-diabetic patients with the use of GLP-1RAs, suggestive of anti-inflammatory and neuroprotective effects. Here we report five real world cases evaluating the impact of GLP-1RA and dual GLP-1/Gastric Inhibitory Polypeptide (GIP) receptor agonist use on Intraocular Pressure (IOP) measurements and subsequent disease management in glaucoma patients.

**Observations:**

Case 1 (severe pigment dispersion glaucoma OU) was started on semaglutide on two separate occasions and exhibited clinically significant declines in home IOP tonometry readings after each initiation (OD max: 20 to 18, 16 to 14; OS: 22 to 15, then 20 to 17). Case 2 (severe primary open angle glaucoma OU) was a candidate for filtration device surgery when he began taking tirzepatide, after which a decrease in home IOP tonometry readings was observed (OD: 11 to 8; OS: 21 to 14), resulting in surgery postponement. Case 3 (secondary glaucoma OD following trauma) began taking tirzepatide shortly following glaucoma drainage device implantation and experienced unexplained hypotony (IOP: 25 to 4). Case 4 (severe POAG OU) was taking semaglutide with stable IOPs until pancreatitis prompted cessation of semaglutide, whereupon an increase in IOP was subsequently observed (OD: 23 to 30; OS: 16 to 18). Case 5 (undiagnosed) is a patient naïve to IOP-lowering therapy and initiation of tirzepatide correlated with an abrupt reduction in IOP observed in clinic (OU: 24 to 10).

**Conclusion and Importance:**

These cases demonstrate a potential association between GLP-1/GIP receptor agonist use and IOP lowering in patients with glaucoma and help substantiate existing literature with real world cases, thereby highlighting the need for further prospective research on this emerging topic.

## Introduction

Glucagon-Like Peptide 1 Receptor Agonists (GLP-1RAs), antidiabetic medications, bind G-protein-coupled receptors in the pancreas increasing insulin secretion in response to oral glucose ingestion. GLP-1 receptors are present in the gastrointestinal system, cardiovascular system and Central Nervous System (CNS) [1]. GLP-1RAs appear to have a role in treating Alzheimer’s, Parkinson’s and stroke due to neuroprotective effects. Mouse models have demonstrated potential neuroprotective effects of GLP-1RAs in the eye and retrospective cohort studies are suggestive of similar effects in patients [2-6].

Although these studies indicate a role for GLP-1RAs in glaucoma prevention, little is known about the impact of GLP-1RAs on IOP in patients. We describe five cases of patients with a glaucoma diagnosis who were prescribed either a GLP-1RA or a dual GLP-1/gastric inhibitory polypeptide (GIP) receptor agonist that suggest a potential IOP-lowering effect of GLP-1RAs.

## Case Report

### Case 1

A 64-year-old white male with severe pigment dispersion glaucoma OU (given VF MD < −12), Obstructive Sleep Apnea (OSA) not on CPAP and controlled hypertension. At diagnosis, clinic IOPs in both eyes (phakic OU) ranged from the mid-20s to high 30s mmHg, with a maximum left eye (OS) IOP of 48 mmHg (Table 1). Management of the patient’s IOP was complicated by his residency abroad and allergic reactions to topical IOP lowering medications. He underwent bilateral Durysta (Allergan plc, Dublin, Ireland) placement in May 2023 and he began measuring IOPs remotely with the iCare HOME2 tonometer (iCare USA, Raleigh, NC) to monitor his response to bilateral Durysta implantation and Selective Laser Trabeculoplasty (SLT) in June 2023.

In October 2023, he presented with iCare HOME2 IOP readings of 10-15 mmHg OD and 10-23 mmHg OS with spikes in the morning hours, suggesting a waning impact of Durysta and SLT. In late October 2023, he began taking semaglutide 5 mg weekly for cardiac dysfunction and concurrently underwent repeat SLT 180 degrees OU. Self-monitoring revealed an IOP decline greater and more immediate than would be expected from SLT to consistently below 15 mmHg OU (Table 1). After a 6-month period, the patient stopped GLP-1RA treatment for over a year. Without any other changes to systemic or ocular therapies, his IOP rose, prompting the provider to consider reinitiation of semaglutide. Notably, he exhibited a similar marked reduction in IOP OU upon GLP-1RA reinitiation.

### Case 2

A 71-year-old white male initially diagnosed with severe normal tension glaucoma in 2003 now severe Primary Open Angle Glaucoma (POAG) OU following SLT x4 OD and x2 OS, a history of Obstructive Sleep Apnea (OSA) on Continuous Positive Airway Pressure (CPAP) and hypertension managed with losartan. The patient reported in June 2020 his IOP increased from a range of 10-13 mmHg in both eyes to 14-15 mmHg OD and 16-17 mmHg OS. Monitoring IOP with an iCare HOME2 tonometer showed increases of ≥ 10 mmHg into the low 20s mmHg OU in the early morning on Rocklatan and Combigan (Table 1). In September 2020, the patient underwent combined cataract surgery and ABiC/Hydrus bilaterally and continued Rocklatan and Combigan OU. By June 2021, becoming intolerant of Rocklatan, he underwent PreserFlo (Santen, Osaka, Japan) OD in January 2022 and resumed Rocklatan OS only, with home tonometry readings of 9-11 mmHg OD and 7-15 mmHg OS. By early and mid-2023, his IOP measurements fluctuated from 8-16 mmHg with a maximum of 21 mmHg OS while OD status post Preserflo was reading 8-11 mmHg off all medications (Table 1).

On October 2, 2023, the patient began tirzepatide for weight loss. His initial dose was 2.5 mg, during which time the patient discontinued Rocklatan and prepared to undergo PreserFlo surgery OS to mitigate the observed IOP elevations. Despite discontinuing the IOP-lowering agent, his IOP remained below 11 mmHg OD and below 14 mmHg OS after December 2023, postponing the need for surgery (Table 1). Both eyes appeared to have a lower IOP and less fluctuation with the initiation of tirzepatide. To date this patient has not undergone additional therapies in either eye due to controlled IOPs in both eyes and remains on his GLP-1RA.

### Case 3

A 44-year-old Hispanic female diagnosed with secondary glaucoma OD following trauma and a history of a prior Penetrating Keratoplasty (PKP) underwent a second PKP of the right eye for graft failure in February 2023. By post-op week 2, her IOP was 25 mmHg OD, decreasing to 17 mmHg by March 2023 with the initiation of Combigan BID. Over the next 3 months, her IOP increased to 45 mmHg by June 2023 (Table 1). Despite addition of Rocklatan, her IOP remained elevated at 37 mmHg in July, at which point she was scheduled for the implantation of a glaucoma drainage device. In August 2023, she underwent an Ahmed ClearPath 250 implantation OD and IOP-lowering agents were paused. On September 15, she began weekly subcutaneous injections of tirzepatide 2.5 mg for pre-diabetes management. Following surgery, her OD IOP ranged from 7-12 mmHg until it increased to 25 mmHg during the hypertensive phase and Combigan once daily was restarted on November 27, 2023. Two weeks later her IOP was suddenly found to be 7 mmHg and her medication regimen was switched to timolol only. In February 2024, her OD IOP was 4 mmHg (Table 1). Shallow choroidals were noted on exam without evidence of over filtration, leakage or a cleft on examination of the anterior chamber and conjunctiva and thus timolol was held. In March and April her hypotony persisted with an IOP between 3-4 mmHg and she was subsequently scheduled for choroidal drainage and tying-off of the glaucoma drainage tube. Throughout this period, her tirzepatide had been gradually titrated up to a maintenance dosage of 10 mg weekly.

### Case 4

A 58-year-old African American female with a history of bilateral quiescent proliferative diabetic retinopathy and severe primary open angle glaucoma OD. Her right eye was at hand-motion vision and underwent a Baervelt-350 implant (Johnson and Johnson Vision, Irvine, CA) in March 2021, with subsequent IOP in the low-20s mmHg on latanoprost, brimonidine tartrate and Cosopt (Table 1). Her less severe left eye underwent SLT x1 and had IOP in the mid-teens on the same regimen and timeline. In June 2022, she started semaglutide 0.25 mg per week for diabetes. Two days later, her IOP was 24 mmHg OD and 15 mmHg OS. Over the following 5 months, her semaglutide dose was titrated up until she reached a maintenance dose of 1.7 mg per week. Her IOP in May 2023 was 23 mmHg OD and 16 mmHg OS (Table 1).

Two months later, she developed severe abdominal pain and was diagnosed with acute pancreatitis, requiring cessation of semaglutide. In November 2023, 4 months post stopping semaglutide, her IOP had increased to 30 mmHg OD and 22 mmHg OS. A fourth agent, netarsudil, was then added for both eyes. Subsequently, her IOP ranged between 20-23 mmHg OD and 13-14 mmHg OS, requiring cyclophotocoagulation OD. Of note, her less severe left eye showed a similar and reversible IOP reduction on semaglutide, decreasing from 19-21 mmHg to 14-16 mmHg and then returning to 20-22 mmHg upon GLP-1RA cessation and without further surgical intervention (Table 1).

### Case 5

A 62-year-old white man with a history of atrial fibrillation and OSA without significant past ocular history was referred in 2018 by his optometrist for narrow angles. His initial IOP was 16 mmHg OD and 15 mmHg OS and his anterior chamber angles were found to be open 360 degrees OU on gonioscopic exam. On imaging of the retinal nerve fiber layer, OS demonstrated borderline thinning of the temporal inferior region. His IOPs were noted to gradually increase, despite stable exam, to a maximum of 24 mmHg at which point cataract extraction was considered. However, prior to any surgery, in February 2022, his IOPs trended back down to his baseline range of 16-18 mmHg OD and 14-16 mmHg OS (Table 1). During his most recent visit in July 2024, the patient’s IOP was the lowest observed to date at 10 mmHg in both eyes, an upwards of 50% drop with no other ocular interventions (Table 1). Notably, the patient had initiated tirzepatide for weight loss in March 2024 at a dose of 2.5 mg that was titrated up to a stable regimen of 7.5 mg in May. He reported a cumulative 40 lbs. of weight loss since initiating treatment.

## Discussion

Clinical impacts of GLP-1RA and GLP-1/GIP receptor dual agonist use in patients with glaucoma or suspected glaucoma are presented. Each patient displayed IOP changes following initiation of GLP-1RAs. Case 1 demonstrates a steady reduction in IOP that was noticeable days after a 180-degree SLT treatment OU. The impact of SLT is generally not expected to fully manifest until several weeks post-procedure, therefore, semaglutide may have contributed to the rapid IOP decrease OU [7]. Subsequently, this patient demonstrated reproducibility of this IOP lowering effect upon reinitiation of the GLP-1RA one year later. Case 2 demonstrated decreased IOP fluctuations within two months of starting the GLP-1RA, despite discontinuation of IOP lowering medication around that same time in the left eye. Case 3 experienced sudden and persistent post-surgical hypotony for six months despite the discontinuation of pressure-lowering drops and without evidence of over-filtration or bleb leak, during which time the dose of tirzepatide was gradually increased. Due to overlap in the postsurgical timing and tirzepatide dose escalation, it is difficult to assign the hypotony to either the medication’s effect on aqueous humor dynamics or an exaggerated post-operative response. Case 4 required an additional IOP-lowering agent to control pressures following the discontinuation of semaglutide and demonstrated reversible IOP reduction in the fellow non-glaucomatous eye. Lastly, after initiating tirzepatide, Case 5 had an observed reduction of IOP OU without the use of IOP-lowering therapies.

These cases suggest IOP-lowering effects of GLP-1RAs and the dual agonist tirzepatide, consistent with preclinical data and supported by mechanism of action [2,8,9]. A published retrospective review with 1,173 patients showed that GLP-1RA use was associated with a modest but statistically significant IOP decrease, an effect more pronounced in glaucoma patients [10].

Several mechanisms of GLP-1RA-mediated IOP lowering and neuroprotection exist for glaucoma. Lawrence, et al., found that in a mouse model of ocular hypertension, topical GLP-1RA liraglutide reduced IOP and rescued Retinal Ganglion Cells (RGCs). Furthermore, neuroinflammatory markers (IL-1a, TNFa and C1q) were downregulated after topical and systemic administration, suggesting a local anti-inflammatory effect independent of its anti-glycemic mechanism [2]. An additional mouse study using an exenatide variant demonstrated lower production of cytokines and cytotoxic astrocytes [3].

GLP-1RAs potentially lower IOP via cAMP- and nitric oxide (NO)-mediated pathways responsible for decreasing aqueous humor secretion and enhanced trabecular outflow, consistent with observed Intracranial Pressure (ICP) reductions in studies using exendin-4 and exenatide [11,12]. Further, GLP-1RAs upregulate NOS expression in multiple tissues, suggesting systemic and ocular NO-mediated vasodilation and IOP reduction [13,14]. Overall, existing literature paints a multifactorial picture of GLP-1RA effects that may be working systemically and ocularly to reduce IOP.

A retrospective study that showed univariate association between GLP-1RAs and reduced IOP did not show statistical significance under multivariate analysis [10]. Cumulatively, other contributing ocular and systemic factors may dilute this association. Our cases highlight specific clinical instances where this association was observed. Moreover, systemic side effects of GLP-1RAs must be considered against potential ocular benefit given the risk for GI discomfort, pancreatitis and gallbladder disease remains relevant. Further, a possible association between GLP-1RA use and NAION demonstrates the need to study how these compounds affect optic nerve perfusion [15,16]. Though these cases alone are insufficient for proving a causal relationship, we believe the collective evidence warrants further investigation and hope that these cases may improve clinician awareness if or when patients experience IOP reductions greater than expected or otherwise unexplainable.

An important consideration in our study is the potentially modifying effect of weight loss. Though several studies report positive correlations between Body Mass Index (BMI) and IOP, a recent analysis of three population-based datasets observed that lower BMI was associated with faster VF progression, greater vertical cup-to-disc ratio and greater likelihood of glaucoma diagnosis [17-21]. GLP-1RA use in non-diabetics was shown to result in a significantly lower risk of POAG and OHTN compared with alternative weight loss therapy at 3- and 5-year intervals, suggestive of an IOP lowering mechanism independent of weight reduction [22].

## Conclusion

Studies in mouse and rat models have shown GLP-1R-mediated neuroprotective and IOP-lowering effects while large retrospective human studies have demonstrated associations between systemic GLP-1RAs, glaucoma incidence and lower IOP. However, the utility of GLP-1RAs for glaucoma patients has not been well established. These five cases suggest an IOP lowering effect of GLP-1RAs, highlighting the need for prospective studies examining the impact of GLP-1RAs on glaucoma diagnosis, pre- and post-surgical outcomes, management and IOP.

## Figures and Tables

**Table 1:** Ophthalmic exam findings and longitudinal Intraocular Pressure (IOP) measurements by case and eye, including cup-to-disc ratio, Central Corneal Thickness (CCT) and maximum IOP (Tmax) across treatment phases relative to GLP-1 receptor agonist use.

Eye	C/D ratio	CCT	Known Tmax	Treated Pre GLP-1 Tmax	On GLP-1 IOP Tmax	Off GLP-1 pre-reinitiation Tmax	On GLP-1 post - reinitiation Tmax
**Case 1**							
OD	0.6	505	36	20	18	16	14
OS	0.85	498	48	22	15	20	17
**Case 2**							
OD	0.9	502	24	11	8	n/a	n/a
OS	0.9	503	25	21	14	n/a	n/a
**Case 3**							
OD	0.3	528	45	25	4	n/a	n/a
OS	0.1	576	n/a	n/a	n/a	n/a	n/a
**Case 4**							
OD	0.9	577	42	24	23	30	n/a
OS	0.85	580	35	15	16	22	n/a
**Case 5**							
OD	0.9	643	24	24	10	n/a	n/a
OS	0.9	645	24	24	10	n/a	n/a

## Data Availability

The data that support the findings of this study are not publicly available due to the nature of the material, which consists of five individual clinical cases. Given the limited sample size and the presence of potentially identifiable clinical details, public data sharing was not deemed feasible or appropriate. However, the data are available from the corresponding author upon reasonable request.
